# Novel eigenvector centrality indices for octane isomers to explore their physicochemical properties

**DOI:** 10.1038/s41598-025-18421-z

**Published:** 2025-10-06

**Authors:** A. Salini Jancy Rani, B. J. Balamurugan

**Affiliations:** Department of Mathematics, School of Advanced Sciences, Vellore Institute of Technology, Chennai Campus, Vandalur-Kelambakkam Road, Chennai, 600127 Tamil Nadu India

**Keywords:** Octane isomers, Eigenvector centrality indices, Quadratic regression, QSPR analysis, Physicochemical properties, Chemistry, Mathematics and computing, Applied mathematics, Computational science, Scientific data, Software, Statistics

## Abstract

In chemical graph theory, a molecular structure is represented as a molecular graph $G(V,E)$, where $V$ denotes the non-empty set of atoms (vertices) and $E$ represents the set of bonds (edges) between the atoms. Centrality measures in a molecular graph are vital for understanding the importance of individual atoms. Among various centrality measures, the eigenvector centrality is a robust metric that captures both the quantity and quality of connections to identify the most influential atoms. Mathematically, the eigenvector centrality $({x}_{i})$ of an atom $i$ in $G(V,E)$ can be defined as the ${i}^{th}$ entry in the normalized eigenvector corresponding to the largest eigenvalue $(\lambda )$ of the adjacency matrix $A\left(G\right)=\left({a}_{ij}\right)$, where ${a}_{ij}=1$ if an atom $i$ is adjacent to an atom $j$ and ${a}_{ij}=0$ otherwise. That is, ${x}_{i}=\frac{1}{\lambda }\sum_{j=1}^{n}{a}_{ij}{x}_{j}$ where $n$ is the number of atoms in $G(V,E)$. In this paper, seven eigenvector centrality-based topological indices are introduced and applied to octane isomers. These indices are utilized in QSPR (Quantitative Structure–Property Relationship) analysis to investigate the properties such as density, mean radius, entropy and more. The results establish a statistically significant and strong correlation between the computed indices and properties of octane isomers. The reliability and accuracy of the regression models are further confirmed through Y-randomization and chi-square goodness-of-fit tests, highlighting the potential of these indices for applications in cheminformatics-based predictive modeling.

## Introduction

Chemical graph theory^1^ in the realms of mathematics and theoretical chemistry, is centered on exploring molecular structures through graph theory principles. This discipline utilizes graph theory concepts to construct molecular graph $G(V,E)$, where $V$ is the non-empty set of vertices and $E$ is the set of edges, such that the graph mirrors the molecular structure by representing each atom as a vertex and each bond as an edge. In this context, the order of the graph $G(V,E)$ refers to the number of atoms in $G(V,E)$, while the size denotes the number of bonds in $G(V,E)$. The degree of an atom $v\in V$, represented as $deg(v)$, is the number of bonds incident to it. An atom $u$ is considered as a neighbor of the atom $v$ if there exists a bond connecting them. The skeletal graph, often referred to as the skeleton graph^2^, is a molecular graph representing the skeletal structure of a molecule. By examining graph representations of molecules, chemical graph theory provides a platform to explore various properties and behaviors, including connectivity, symmetry, stability, and reactivity. A notable method for analyzing graph representations involves the use of topological indices, whose origins can be traced back to the pioneering work of H. Wiener in 1947^3^. A topological index (TI) of a molecular graph $G$ is a numerical representation that incorporates graph-theoretic metrics such as vertex degree (the number of connections a vertex has), distance (the length of the shortest path) between vertices, or other graph invariants that encode topological information about the molecular structure^1^. Topological indices offer predictive insights into the chemical, pharmacological, and biological facets of chemical substances through the Quantitative Structure–Property Relationship (QSPR) analysis. This approach empowers researchers to predict and investigate the physicochemical attributes of novel molecules endowed with known properties. For instance, W. Tamilarasi et al.^4^ employed the QSPR analysis to predict ADMET properties of anti-COVID drugs against the Omicron variant using several degree-based topological indices. For a more in-depth understanding of QSPR analysis, refer to sources^5–11^.

Many researchers opt for degree-based topological indices in their studies due to their computational simplicity^12–17^. These indices focus solely on the degree, representing the quantity of connections for an atom. However, it is possible to enhance the robustness of an index value by considering not only the quantity but also the quality of connections for an atom. In this regard, eigenvector centrality of atom serves this purpose. P. Bonacich^18^ introduced the concept of eigenvector centrality of vertices (atoms) in 1987, relying on eigenvector principles from linear algebra. This metric finds application across diverse disciplines like social network analysis, biology, and computer science for pinpointing influential or noteworthy vertices in a network. In a study, L. Lorenzini et al.^19^ suggested that eigenvector centrality could serve as an early biomarker of the relationship between the onset of Alzheimer’s disease pathology and cognitive decline. Similarly, S. Skouras et al. ^20^ explored eigenvector centrality mapping on the neural mechanisms of functional compensation across the pathophysiological continuum of Alzheimer’s disease, highlighting the compensatory roles of several key brain areas. G. Lohmann et al.^21^ harnessed eigenvector centrality to map connectivity patterns in fMRI data of human brain. Moon et al.^22^ utilized eigenvector centrality to identify the most common acupoint combinations for Functional Dyspepsia and Irritable Bowel Syndrome. Additionally, C.F.A. Negre et al.^23^ used the eigenvector centrality measure for the characterization of protein allosteric pathways.

In the field of organic chemistry^24^, structural isomers (referred to as constitutional isomers in the nomenclature of the International Union of Pure and Applied Chemistry) are molecules with the same molecular composition, which precisely mirrors the count of atoms for each element therein but differs in the configuration of chemical bonds that connect these elements. Octane $({C}_{8}{H}_{18})$, a hydrocarbon and specifically an alkane, has eighteen structural isomers, each with disparate physical and chemical characteristics. Researchers widely use octane isomers as test compounds to investigate newly introduced topological indices in QSPR analysis^25–31^. In this paper, eigenvector centrality is computed to develop novel topological indices with the primary objective of investigating their ability to explore the properties of chemical compounds. Eigenvector centrality-based indices for the skeletal graphs of all the eighteen octane isomers are computed. Subsequently, these indices are utilized in QSPR analysis to explore the properties of octane isomers, such as density $(D)$, mean radius $({R}_{m}^{2})$, entropy $(S)$, acentric factor $(AF$), standard enthalpy of vaporization $(DHVAP)$, boiling point $(BP)$, enthalpy of vaporization $({H}_{v})$, enthalpy of formation $({H}_{f})$, enthalpy of combustion $({H}_{c})$ and critical pressure $({P}_{c})$, through quadratic regression. The findings reveal a significant and strong correlation between the computed indices and properties being investigated. The predictive performance of the regression models is validated using Y-randomization and chi-square goodness-of-fit tests, confirming their robustness and accuracy.

## Concepts and terminologies

Alkanes are a class of hydrocarbons, which are organic compounds composed solely of carbon $(C)$ and hydrogen $(H)$ atoms. Their key feature is the presence of single bonds linking the carbon atoms, rendering them saturated hydrocarbons. The general formula for alkanes is ${C}_{n}{H}_{2n+2}$, where $n$ is the number of carbon atoms in the molecule.

Isomers are compounds that have the same molecular formula but different structural formulas. They contain the same number and types of atoms but differ in the connectivity of these atoms, leading to different physical and chemical properties for each isomer.

The skeletal structure of an alkane is a structure that shows the carbon–carbon bonds as lines but does not show the hydrogens bonded to the carbons, as each carbon is understood to be bonded to the appropriate number of hydrogens to satisfy its valency of four bonds. This simplified representation focuses solely on the carbon framework of the molecule.

The skeletal graph of a molecule is a molecular graph that provides a simplified graphical representation of its structure, mirroring its skeletal formula by portraying carbon atoms as vertices and chemical bonds between the carbon atoms as edges.

### Definitions

#### Definition 2.1.1

Let $G(V,E)$ be a molecular graph with order $n$ and size $m$. The adjacency matrix $A(G)$ of the molecular graph $G$ is a $n\times n$ symmetric binary matrix, which can be defined as.

$$A(G)=\left({a}_{ij}\right)=\left\{\begin{array}{c}1, if\ i\ and\ j\ are\ adjacent\ atoms \\ 0, otherwise\hspace{3.7cm}\end{array}\right.$$

#### Definition 2.1.2

The eigenvector centrality $({x}_{i})$ of an atom $i$ in a molecular graph $G(V,E)$ is defined as the ${i}^{th}$ entry in the normalized eigenvector corresponding to the largest eigenvalue $(\lambda )$ of $A(G)$. That is,

$${x}_{i}=\frac{1}{\lambda }\sum_{j=1}^{n}{a}_{ij}{x}_{j}=\frac{1}{\lambda }\sum_{j\in N(i)}{x}_{j}$$

where $n$ is the total number of atoms and $N(i)$ is the set of the neighbors of $i$.

In the field of matrix theory, the Perron-Frobenius theorem^32^, established by Oskar Perron and Georg Frobenius, affirms that a square matrix containing non-negative real elements has a unique largest real eigenvalue whose corresponding eigenvectors are strictly positive. Since the adjacency matrix of any molecular graph is a square matrix containing non-negative real entries, the Perron-Frobenius theorem guarantees that the eigenvector centrality values are strictly positive.

## Eigenvector centrality-based indices of molecular graphs

Eigenvector centrality of an atom provides a numerical description of how well that atom is connected to other highly connected atoms, making it a superior metric for analyzing molecular graphs compared to degree, closeness, or betweenness centrality. This global perspective aligns well with molecular systems, where an atom’s influence extends beyond its immediate neighbors to the neighbors of those neighbors. Motivated by this inherent property, this work proposes a set of novel topological indices based on eigenvector centrality to enhance the structural representation of molecules in QSPR modeling.

For a molecular graph $G$, the newly proposed indices are designated as the First eigenvector centrality index ${M}_{{1}_{x}}(G)$, Second eigenvector centrality index ${M}_{{2}_{x}}(G)$, Forgotten eigenvector centrality index ${F}_{x}(G)$, Harmonic eigenvector centrality index ${H}_{x}(G)$, Inverse sum eigenvector centrality index $I{S}_{x}(G)$, Reciprocal Randić eigenvector centrality index $R{R}_{x}(G)$ and Atom-bond connectivity eigenvector centrality index $AB{C}_{x}(G)$ and these indices are defined as follows.

$${M}_{{1}_{x}}\left(G\right)=\sum_{ij\in E\left(G\right)}[{x}_{i}+{x}_{j}]$$

$${M}_{{2}_{x}}\left(G\right)=\sum_{ij\in E(G)}{x}_{i}.{x}_{j}$$

$${F}_{x}\left(G\right)=\sum_{ij\in E(G)}[{x}_{i}^{2}+{x}_{j}^{2}]$$

$${H}_{x}\left(G\right)=\sum_{ij\in E(G)}\frac{2}{{x}_{i}+{x}_{j}}$$

$$I{S}_{x}\left(G\right)=\sum_{ij\in E(G)}\frac{{x}_{i}.{x}_{j}}{{x}_{i}+{x}_{j}}$$

$$R{R}_{x}\left(G\right)=\sum_{ij\in E(G)}\sqrt{{x}_{i}.{x}_{j}}$$

$$AB{C}_{x}\left(G\right)=\sum_{ij\in E(G)}\sqrt{\left|\frac{{x}_{i}+{x}_{j}-2}{{x}_{i}.{x}_{j}}\right|}$$

## Eigenvector centrality-based indices of octane isomers

Octane is a hydrocarbon with the molecular formula $${C}_{8}{H}_{18}$$ and belongs to the alkane family. It has eighteen isomers and is commonly used as a reference standard in the gasoline industry. Chemists often use skeletal structures of octane isomers due to their compactness and ability to retain structural information. Octane isomers have become a key group of organic molecules for evaluating various topological parameters in QSPR. Therefore, this study investigates the effectiveness of newly introduced eigenvector centrality-based indices in exploring the physicochemical properties of chemical structures, specifically using the skeletal structures of octane isomers. Considering $$G(V,E)$$ as the skeletal graph of octane isomers with the atom set $$V$$ and the bond set $$E$$, the eigenvector centrality-based indices $$({M}_{{1}_{x}}, {M}_{{2}_{x}}$$, $${F}_{x}$$, $${H}_{x}$$, $$I{S}_{x}$$, $$R{R}_{x}$$ and $$AB{C}_{x})$$ are computed for all the octane isomers. Note that the skeletal graph $$G$$ of each isomer, $$\left|V(G)\right|=8$$ and $$\left|E(G)\right|=7$$.

### Theorem 4.1

*Let* ${G}_{1}(V,E)$ *be the skeletal graph of* $n$-*octane with the atom set* $V$ *and bond set* $E$. *Then the eigenvector centrality-based topological indices of the skeletal graph* ${G}_{1}$ *are* ${{M}_{1}}_{x}({G}_{1})=5.0240$,${{M}_{2}}_{x}({G}_{1})=0.9395$,${F}_{x}({G}_{1})=1.9476$,${H}_{x}({G}_{1})=20.9806$,$I{S}_{x}\left({G}_{1}\right)=1.2247$, $R{R}_{x}({G}_{1})=2.4801$ *and* $AB{C}_{x}({G}_{1})=24.7841$.

### Proof

Let ${G}_{1}(V,E)$ be the skeletal graph of $n$-octane with $|V({G}_{1})| = 8$ and $|E({G}_{1})| = 7$. The skeletal structure of $n$-octane and its corresponding skeletal graph are shown in Table 1. The adjacency matrix of ${G}_{1}$ is

**Table 1:** Eigenvector centrality value $({x}_{i})$ of each atom $i$ in the skeletal graph of $n$-octane.

		
	**(a)** Skeletal structure of $n$-octane				**(b)** Skeletal graph of $n$-octane			
$i$	1	2	3	4	5	6	7	8
${x}_{i}$	0.1612	0.3030	0.4082	0.4642	0.4642	0.4082	0.3030	0.1612

$$\text{A}({\text{G}}_{1})=\left(\begin{array}{cccccccc}0& 1& 0& 0& 0& 0& 0& 0\\ 1& 0& 1& 0& 0& 0& 0& 0\\ 0& 1& 0& 1& 0& 0& 0& 0\\ 0& 0& 1& 0& 1& 0& 0& 0\\ 0& 0& 0& 1& 0& 1& 0& 0\\ 0& 0& 0& 0& 1& 0& 1& 0\\ 0& 0& 0& 0& 0& 1& 0& 1\\ 0& 0& 0& 0& 0& 0& 1& 0\end{array}\right)$$

The eigenvector centrality values $({x}_{i})$ for the atoms $(i)$ of ${G}_{1}$ are shown in Table 1. These values are computed from the adjacency matrix $A({G}_{1})$ through MATLAB programming.

$\left(i\right)$ The first eigenvector centrality index ${M}_{{1}_{x}}\left({G}_{1}\right)$ is

$$\begin{gathered} M_{{1_{x} }} \left( {G_{1} } \right) = \mathop \sum \limits_{{ij \in E\left( {G_{1} } \right)}} [x_{i} + x_{j} ] \hfill \\ = \left( {x_{1} + x_{2} } \right) + \left( {x_{2} + x_{3} } \right) + \left( {x_{3} + x_{4} } \right) + \left( {x_{4} + x_{5} } \right) + \left( {x_{5} + x_{6} } \right) + \left( {x_{6} + x_{7} } \right) + \left( {x_{7} + x_{8} } \right) \hfill \\ = \left( {0.1612 + 0.3030} \right) + \left( {0.3030 + 0.4082} \right) + \left( {0.4082 + 0.4642} \right) \hfill \\ + \left( {0.4642 + 0.4642} \right) + \left( {0.4642 + 0.4082} \right) + \left( {0.4082 + 0.3030} \right) \hfill \\ + \left( {0.3030 + 0.1612} \right) = 5.0240 \hfill \\ \end{gathered}$$

$\left(ii\right)$ The second eigenvector centrality index ${M}_{{2}_{x}}\left({G}_{1}\right)$ is

$$\begin{gathered} M_{{2_{x} }} \left( {G_{1} } \right) = \mathop \sum \limits_{{ij \in E\left( {G_{1} } \right)}} x_{i} \cdot x_{j} \hfill \\ = \left( {x_{1} \cdot x_{2} } \right) + \left( {x_{2} \cdot x_{3} } \right) + \left( {x_{3} \cdot x_{4} } \right) + \left( {x_{4} \cdot x_{5} } \right) + \left( {x_{5} \cdot x_{6} } \right) + \left( {x_{6} \cdot x_{7} } \right) + \left( {x_{7} \cdot x_{8} } \right) \hfill \\ = \left( {0.1612 \times 0.3030} \right) + \left( {0.3030 \times 0.4082} \right) + \left( {0.4082 \times 0.4642} \right) + \left( {0.4642 \times 0.4642} \right) \hfill \\ + \left( {0.4642 \times 0.4082} \right) + \left( {0.4082 \times 0.3030} \right) + \left( {0.3030 \times 0.1612} \right) = 0.9395 \hfill \\ \end{gathered}$$

$\left(iii\right)$ Forgotten eigenvector centrality index ${F}_{x}\left({G}_{1}\right)$ is

$$\begin{gathered} F_{x} \left( {G_{1} } \right) = \mathop \sum \limits_{{ij \in E\left( {G_{1} } \right)}} [x_{i}^{2} + x_{j}^{2} ] \hfill \\ = \left( {x_{1}^{2} + x_{2}^{2} } \right) + \left( {x_{2}^{2} + x_{3}^{2} } \right) + \left( {x_{3}^{2} + x_{4}^{2} } \right) + \left( {x_{4}^{2} + x_{5}^{2} } \right) + \left( {x_{5}^{2} + x_{6}^{2} } \right) + \left( {x_{6}^{2} + x_{7}^{2} } \right) + \left( {x_{7}^{2} + x_{8}^{2} } \right) \hfill \\ = \left( {0.1612^{2} + 0.3030^{2} } \right) + \left( {0.3030^{2} + 0.4082^{2} } \right) + \left( {0.4082^{2} + 0.4642^{2} } \right) \hfill \\ + \left( {0.4642^{2} + 0.4642^{2} } \right) + \left( {0.4642^{2} + 0.4082^{2} } \right) + \left( {0.4082^{2} + 0.3030^{2} } \right) \hfill \\ + \left( {0.3030^{2} + 0.1612^{2} } \right) = 1.9476 \hfill \\ \end{gathered}$$

$\left(iv\right)$ Harmonic eigenvector centrality index ${H}_{x}\left({G}_{1}\right)$ is

$$\begin{gathered} H_{x} \left( {G_{1} } \right) = \mathop \sum \limits_{{ij \in E\left( {G_{1} } \right)}} \frac{2}{{x_{i} + x_{j} }} \hfill \\ = \frac{2}{{x_{1} + x_{2} }} + \frac{2}{{x_{2} + x_{3} }} + \frac{2}{{x_{3} + x_{4} }} + \frac{2}{{x_{4} + x_{5} }} + \frac{2}{{x_{5} + x_{6} }} + \frac{2}{{x_{6} + x_{7} }} + \frac{2}{{x_{7} + x_{8} }} \hfill \\ = \frac{2}{0.1612 + 0.3030} + \frac{2}{0.3030 + 0.4082} + \frac{2}{0.4082 + 0.4642} + \frac{2}{0.4642 + 0.4642} \hfill \\ + \frac{2}{0.4642 + 0.4082} + \frac{2}{0.4082 + 0.3030} + \frac{2}{0.3030 + 0.1612} = 20.9806 \hfill \\ \end{gathered}$$

$\left(v\right)$ Inverse sum eigenvector centrality index ${IS}_{x}\left({G}_{1}\right)$ is

$$\begin{gathered} IS_{x} \left( {G_{1} } \right) = \mathop \sum \limits_{{ij \in E\left( {G_{1} } \right)}} \frac{{x_{i} .x_{j} }}{{x_{i} + x_{j} }} \hfill \\ = \frac{{x_{1} .x_{2} }}{{x_{1} + x_{2} }} + \frac{{x_{2} .x_{3} }}{{x_{2} + x_{3} }} + \frac{{x_{3} .x_{4} }}{{x_{3} + x_{4} }} + \frac{{x_{4} .x_{5} }}{{x_{4} + x_{5} }} + \frac{{x_{5} .x_{6} }}{{x_{5} + x_{6} }} + \frac{{x_{6} .x_{7} }}{{x_{6} + x_{7} }} + \frac{{x_{7} .x_{8} }}{{x_{7} + x_{8} }} \hfill \\ = \frac{0.1612 \times 0.3030}{{0.1612 + 0.3030}} + \frac{0.3030 \times 0.4082}{{0.3030 + 0.4082}} + \frac{0.4082 \times 0.4642}{{0.4082 + 0.4642}} + \frac{0.4642 \times 0.4642}{{0.4642 + 0.4642}} \hfill \\ + \frac{0.4642 \times 0.4082}{{0.4642 + 0.4082}} + \frac{0.4082 \times 0.3030}{{0.4082 + 0.3030}} + \frac{0.3030 \times 0.1612}{{0.3030 + 0.1612}} = 1.2247 \hfill \\ \end{gathered}$$

$\left(vi\right)$ Reciprocal Randić eigenvector centrality index ${RR}_{x}\left({G}_{1}\right)$ is

$$\begin{gathered} RR_{x} \left( {G_{1} } \right) = \mathop \sum \limits_{{ij \in E\left( {G_{1} } \right)}} \sqrt {x_{i} .x_{j} } \hfill \\ = \sqrt {x_{1} .x_{2} } + \sqrt {x_{2} .x_{3} } + \sqrt {x_{3} .x_{4} } + \sqrt {x_{4} .x_{5} } + \sqrt {x_{5} .x_{6} } + \sqrt {x_{6} .x_{7} } + \sqrt {x_{7} .x_{8} } \hfill \\ = \sqrt {0.1612 \times 0.3030} + \sqrt {0.3030 \times 0.4082} + \sqrt {0.4082 \times 0.4642} + \sqrt {0.4642 \times 0.4642} \hfill \\ + \sqrt {0.4642 \times 0.4082} + \sqrt {0.4082 \times 0.3030} + \sqrt {0.3030 \times 0.1612} = 2.4801 \hfill \\ \end{gathered}$$

$\left(vii\right)$ Atom-bond connectivity eigenvector centrality index $AB{C}_{x}\left({G}_{1}\right)$ is

$$\begin{gathered} ABC_{x} \left( {G_{1} } \right) = \mathop \sum \limits_{{ij \in E\left( {G_{1} } \right)}} \sqrt {\left| {\frac{{x_{i} + x_{j} - 2}}{{x_{i} .x_{j} }}} \right|} \hfill \\ = \sqrt {\left| {\frac{{x_{1} + x_{2} - 2}}{{x_{1} .x_{2} }}} \right|} + \sqrt {\left| {\frac{{x_{2} + x_{3} - 2}}{{x_{2} .x_{3} }}} \right|} + \sqrt {\left| {\frac{{x_{3} + x_{4} - 2}}{{x_{3} .x_{4} }}} \right|} + \sqrt {\left| {\frac{{x_{4} + x_{5} - 2}}{{x_{4} .x_{5} }}} \right|} \hfill \\ + \sqrt {\left| {\frac{{x_{5} + x_{6} - 2}}{{x_{5} .x_{6} }}} \right|} + \sqrt {\left| {\frac{{x_{6} + x_{7} - 2}}{{x_{6} .x_{7} }}} \right|} + \sqrt {\left| {\frac{{x_{7} + x_{8} - 2}}{{x_{7} .x_{8} }}} \right|} \hfill \\ \user2{ } = \sqrt {\left| {\frac{0.1612 + 0.3030 - 2}{{0.1612 \times 0.3030}}} \right|} + \sqrt {\left| {\frac{0.3030 + 0.4082 - 2}{{0.3030 \times 0.4082}}} \right|} \hfill \\ + \sqrt {\left| {\frac{0.4082 + 0.4642 - 2}{{0.4082 \times 0.4642}}} \right|} + \sqrt {\left| {\frac{0.4642 + 0.4642 - 2}{{0.4642 \times 0.4642}}} \right|} \hfill \\ + \sqrt {\left| {\frac{0.4642 + 0.4082 - 2}{{0.4642 \times 0.4082}}} \right|} + \sqrt {\left| {\frac{0.4082 + 0.3030 - 2}{{0.4082 \times 0.3030}}} \right|} \hfill \\ + \sqrt {\left| {\frac{0.3030 + 0.1612 - 2}{{0.3030 \times 0.1612}}} \right|} = 24.7841 \hfill \\ \end{gathered}$$

Thus, the eigenvector centrality indices for the skeletal graph of $n$- octane are obtained.□

The skeletal structures of all the octane isomers are obtained from ChemSpider database. The skeletal structures and their corresponding skeletal graphs of the remaining seventeen octane isomers viz., 2-methylheptane, 3-methylheptane, 4-methylheptane, 3-ethylhexane, 2,2-dimethylhexane, 2,3-dimethylhexane, 2,4-dimethylhexane, 2,5-dimethylhexane, 3,3-dimethylhexane, 3,4-dimethylhexane, 3-ethyl-2-methylpentane, 3-ethyl-3-methylpentane, 2,2,3-trimethylpentane, 2,2,4-trimethylpentane, 2,3,3-trimethylpentane, 2,3,4-trimethylpentane and 2,2,3,3-tetramethylbutane are presented in Tables 2, 3, 4, 5, 6, 7, 8, 9, 10, 11, 12, 13, 14, 15, 16, 17 and 18. The eigenvector centrality values $$({x}_{i})$$ assigned to each atom $$i$$ of all these octane isomers are computed through MATLAB programming from the adjacency matrix of their corresponding skeletal graphs and are included in these tables. The eigenvector centrality-based indices $$({M}_{{1}_{x}}, {M}_{{2}_{x}}$$, $${F}_{x}$$, $${H}_{x}$$, $$I{S}_{x}$$, $$R{R}_{x}$$ and $$AB{C}_{x})$$ for the remaining seventeen isomers are calculated in the same way as in the Theorem 4.1 and are shown in Table 19.

**Table 2:** Eigenvector centrality value $({x}_{i})$ of each atom $i$ in the skeletal graph of 2-methylheptane.

		
	**(a)** Skeletal structure of 2-methylheptane				**(b)** Skeletal graph of 2-methylheptane			
$i$	1	2	3	4	5	6	7	8
${x}_{i}$	0.2673	0.5211	0.4816	0.4179	0.3333	0.2319	0.1189	0.2673

**Table 3:** Eigenvector centrality value $({x}_{i})$ of each atom $i$ in the skeletal graph of 3-methylheptane.

		
	**(a)** Skeletal structure of 3-methylheptane				**(b)** Skeletal graph of 3-methylheptane			
$i$	1	2	3	4	5	6	7	8
${x}_{i}$	0.1927	0.3834	0.5698	0.4635	0.3521	0.2369	0.1191	0.2865

**Table 4:** Eigenvector centrality value $({x}_{i})$ of each atom $i$ in the skeletal graph of 4-methylheptane.

		
	**(a)** Skeletal structure of 4-methylheptane				**(b)** Skeletal graph of 4-methylheptane			
$i$	1	2	3	4	5	6	7	8
${x}_{i}$	0.1443	0.2887	0.4330	0.5774	0.4330	0.2887	0.1443	0.2887

**Table 5:** Eigenvector centrality value $({x}_{i})$ of each atom $i$ in the skeletal graph of 3-ethylhexane.

		
	**(a)** Skeletal structure of 3-ethylhexane				**(b)** Skeletal graph of 3-ethylhexane			
$i$	1	2	3	4	5	6	7	8
${x}_{i}$	0.1899	0.3851	0.5914	0.4294	0.2796	0.1378	0.3851	0.1899

**Table 6:** Eigenvector centrality value $({x}_{i})$ of each atom $i$ in the skeletal graph of 2,2-dimethylhexane.

		
	**(a)** Skeletal structure of 2,2-dimethylhexane				**(b)** Skeletal graph of 2,2-dimethylhexane			
$i$	1	2	3	4	5	6	7	8
${x}_{i}$	0.3019	0.6376	0.4409	0.2936	0.1792	0.0849	0.3019	0.3019

**Table 7:** Eigenvector centrality value $({x}_{i})$ of each atom $i$ in the skeletal graph of 2,3-dimethylhexane.

		
	**(a)** Skeletal structure of 2,3-dimethylhexane				**(b)** Skeletal graph of 2,3-dimethylhexane			
$i$	1	2	3	4	5	6	7	8
${x}_{i}$	0.2454	0.5090	0.5651	0.3907	0.2454	0.1183	0.2454	0.2724

**Table 8:** Eigenvector centrality value $({x}_{i})$ of each atom $i$ in the skeletal graph of 2,4-dimethylhexane.

		
	**(a)** Skeletal structure of 2,4-dimethylhexane				**(b)** Skeletal graph of 2,4-dimethylhexane			
$i$	1	2	3	4	5	6	7	8
${x}_{i}$	0.2188	0.4467	0.4747	0.5227	0.3367	0.1649	0.2188	0.2560

**Table 9:** Eigenvector centrality value $({x}_{i})$ of each atom $i$ in the skeletal graph of 2,5-dimethylhexane.

		
	**(a)** Skeletal structure of 2,5-dimethylhexane				**(b)** Skeletal graph of 2,5-dimethylhexane			
$i$	1	2	3	4	5	6	7	8
${x}_{i}$	0.2236	0.4472	0.4472	0.4472	0.4472	0.2236	0.2236	0.2236

**Table 10:** Eigenvector centrality value $({x}_{i})$ of each atom $i$ in the skeletal graph of 3,3-dimethylhexane.

		
	**(a)** Skeletal structure of 3,3-dimethylhexane				**(b)** Skeletal graph of 3,3-dimethylhexane			
$i$	1	2	3	4	5	6	7	8
${x}_{i}$	0.1755	0.3786	0.6409	0.4093	0.2417	0.1121	0.2972	0.2972

**Table 11:** Eigenvector centrality value $({x}_{i})$ of each atom $i$ in the skeletal graph of 3,4-dimethylhexane.

		
	**(a)** Skeletal structure of 3,4-dimethylhexane				**(b)** Skeletal graph of 3,4-dimethylhexane			
$i$	1	2	3	4	5	6	7	8
${x}_{i}$	0.1601	0.3355	0.5428	0.5428	0.3355	0.1601	0.2591	0.2591

**Table 12:** Eigenvector centrality value $({x}_{i})$ of each atom $i$ in the skeletal graph of 3-ethyl-2-methylpentane.

		
	**(a)** Skeletal structure of 3-ethyl-2-methylpentane				**(b)** Skeletal graph of 3-ethyl-2-methylpentane			
$i$	1	2	3	4	5	6	7	8
${x}_{i}$	0.2380	0.5000	0.5745	0.3536	0.1683	0.2380	0.3536	0.1683

**Table 13:** Eigenvector centrality value $({x}_{i})$ of each atom $i$ in the skeletal graph of 3-ethyl-3-methylpentane.

		
	**(a)** Skeletal structure of 3-ethyl-3-methylpentane				**(b)** Skeletal graph of 3-ethyl-3-methylpentane			
$i$	1	2	3	4	5	6	7	8
${x}_{i}$	0.1696	0.3713	0.6432	0.3713	0.1696	0.2938	0.3713	0.1696

**Table 14:** Eigenvector centrality value $({x}_{i})$ of each atom $i$ in the skeletal graph of 2,2,3-trimethylpentane.

		
	**(a)** Skeletal structure of 2,2,3-trimethylpentane				**(b)** Skeletal graph of 2,2,3-trimethylpentane			
$i$	1	2	3	4	5	6	7	8
${x}_{i}$	0.2729	0.6019	0.5092	0.2905	0.1317	0.2729	0.2729	0.2308

**Table 15:** Eigenvector centrality value $({x}_{i})$ of each atom $i$ in the skeletal graph of 2,2,4-trimethylpentane.

		
	**(a)** Skeletal structure of 2,2,4-trimethylpentane				**(b)** Skeletal graph of 2,2,4-trimethylpentane			
$i$	1	2	3	4	5	6	7	8
${x}_{i}$	0.2799	0.6015	0.4529	0.3717	0.1730	0.2799	0.2799	0.1730

**Table 16:** Eigenvector centrality value $({x}_{i})$ of each atom $i$ in the skeletal graph of 2,3,3-trimethylpentane.

		
	**(a)** Skeletal structure of 2,3,3-trimethylpentane				**(b)** Skeletal graph of 2,3,3-trimethylpentane			
$i$	1	2	3	4	5	6	7	8
${x}_{i}$	0.2115	0.4700	0.6210	0.3505	0.1578	0.2115	0.2795	0.2795

**Table 17:** Eigenvector centrality value $({x}_{i})$ of each atom $i$ in the skeletal graph of 2,3,4-trimethylpentane.

		
	**(a)** Skeletal structure of 2,3,4-trimethylpentane				**(b)** Skeletal graph of 2,3,4-trimethylpentane			
$i$	1	2	3	4	5	6	7	8
${x}_{i}$	0.2176	0.4647	0.5573	0.4647	0.2176	0.2176	0.2610	0.2176

**Table 18:** Eigenvector centrality value $({x}_{i})$ of each atom $i$ in the skeletal graph of 2,2,3,3-tetramethyl-butane.

		
	**(a)** Skeletal structure of 2,2,3,3-tetramethylbutane				**(b)** Skeletal graph of 2,2,3,3-tetramethylbutane			
$i$	1	2	3	4	5	6	7	8
${x}_{i}$	0.2454	0.5651	0.5651	0.2454	0.2454	0.2454	0.2454	0.2454

**Table 19:** Eigenvector centrality-based indices of octane isomers.

Structural isomers of octane	${{M}_{1}}_{x}$	${{M}_{2}}_{x}$	${F}_{x}$	${H}_{x}$	${IS}_{x}$	${RR}_{x}$	${ABC}_{x}$
$n$-octane	5.0240	0.9395	1.9476	20.9806	1.2247	2.4801	24.7841
2-methylheptane	5.1462	0.9751	2.1146	21.1939	1.2284	2.5135	25.2623
3-methylheptane	5.1795	0.9945	2.1913	21.3067	1.2246	2.5179	25.5812
4-methylheptane	5.1963	1.0001	2.2085	21.0484	1.2263	2.5238	25.2431
3-ethylhexane	5.2502	1.0141	2.2587	20.6245	1.2310	2.5418	24.7245
2,2-dimethylhexane	5.3621	1.0553	2.5322	22.9518	1.2198	2.5567	28.2542
2,3-dimethylhexane	5.3760	1.0370	2.3696	20.2880	1.2442	2.5853	24.3161
2,4-dimethylhexane	5.3895	1.0208	2.2842	19.0692	1.2597	2.6046	22.4204
2,5-dimethylhexane	5.3664	1.0000	2.1999	18.6345	1.2671	2.6066	21.6368
3,3-dimethylhexane	5.5048	1.0783	2.6016	20.4646	1.2422	2.6144	24.7889
3,4-dimethylhexane	5.4372	1.0474	2.4036	19.4557	1.2534	2.6096	23.1497
3-ethyl-2-methylpentane	5.4505	1.0505	2.4102	19.2556	1.2556	2.6151	22.8709
3-ethyl-3-methylpentane	5.6032	1.0944	2.6546	19.1413	1.2573	2.6538	22.9035
2,2,3-trimethylpentane	5.6974	1.1031	2.6898	18.5995	1.2737	2.6927	22.0957
2,2,4-trimethylpentane	5.6126	1.0745	2.5668	18.4731	1.2717	2.6704	21.7526
2,3,3-trimethylpentane	5.7348	1.1109	2.7216	18.1379	1.2777	2.7059	21.4494
2,3,4-trimethylpentane	5.5915	1.0679	2.4849	18.0830	1.2774	2.6711	21.1771
2,2,3,3-tetramethylbutane	5.9932	1.1515	2.9160	16.5753	1.3093	2.7996	19.2214

## Results and discussion

### QSPR analysis of octane isomers for physicochemical properties

It is a standard practice to use a group of alkanes as test compounds, focusing particularly on the eighteen octane isomers in comparative tests to verify the accuracy of statistical results. The experimental values of density $(D)$, mean radius $({R}_{m}^{2})$, entropy $(S)$, acentric factor $(AF$), standard enthalpy of vaporization $(DHVAP)$, boiling point $(BP)$, enthalpy of vaporization $({H}_{v})$, enthalpy of formation $({H}_{f})$, enthalpy of combustion $({H}_{c})$ and critical pressure $({P}_{c})$ of octane isomers are gathered from ChemSpider database and shown in Tables 20 and 21. The QSPR analysis is carried out between the theoretically computed indices and the experimental values of the octane isomers through quadratic regression to analyze their physicochemical properties.

**Table 20:** Experimental values of physicochemical properties $(D, {R}_{m}^{2}, S, AF, DHVAP)$ of octane isomers.

Structural isomers of octane	$D$ $(g/c{m}^{3})$	${R}_{m}^{2}$ (Å^2^)	$S$ $(J/(mol\cdot K))$	$AF$ $(-)$	$DHVAP$ $(KJ/mol)$
$n$-octane	0.7025	2.0449	111.67	0.3979	9.915
2-methylheptane	0.6980	1.8913	109.84	0.3779	9.484
3-methylheptane	0.7058	1.7984	111.26	0.3710	9.521
4-methylheptane	0.7046	1.7673	109.32	0.3715	9.483
3-ethylhexane	0.7136	1.7673	109.43	0.3625	9.476
2,2-dimethylhexane	0.6953	1.6744	103.42	0.3394	8.915
2,3-dimethylhexane	0.7121	1.6464	108.02	0.3483	9.272
2,4-dimethylhexane	0.7004	1.6142	106.98	0.3442	9.029
2,5-dimethylhexane	0.6935	1.6449	105.72	0.3568	9.051
3,3-dimethylhexane	0.7100	1.7377	104.74	0.3226	8.973
3,4-dimethylhexane	0.7200	1.5230	106.59	0.3404	9.316
3-ethyl-2-methylpentane	0.7193	1.5525	106.06	0.3324	9.209
3-ethyl-3-methylpentane	0.7274	1.5214	101.48	0.3069	9.081
2,2,3-trimethylpentane	0.7161	1.4306	101.31	0.3008	8.826
2,2,4-trimethylpentane	0.6919	1.4010	104.09	0.3054	8.402
2,3,3-trimethylpentane	0.7262	1.4931	102.06	0.2932	8.897
2,3,4-trimethylpentane	0.7191	1.3698	102.39	0.3174	9.014
2,2,3,3-tetramethylbutane	0.8242	1.4612	93.06	0.2553	8.410

**Table 21:** Experimental values of physicochemical properties $(BP, {H}_{v}, {H}_{f}, {H}_{c}, {P}_{c})$ of octane isomers.

Structural isomers of octane	$BP$ $(^\circ C)$	${H}_{v}$ $(KJ/mol)$	${H}_{f}$ $(KJ/mol)$	${H}_{c}$ $(KJ/mol)$	${P}_{c}$ $(MPa)$
$n$-octane	125.665	73.19	− 49.82	1222.77	24.54
2-methyl-heptane	117.647	70.30	− 51.50	1221.08	24.52
3-methyl-heptane	118.925	71.30	− 50.82	1221.76	25.13
4-methyl-heptane	117.709	70.91	− 50.69	1221.89	25.09
3-ethyl-hexane	118.534	71.70	− 50.40	1222.19	25.74
2,2-dimethyl-hexane	106.840	67.70	− 53.71	1218.88	24.76
2,3-dimethyl-hexane	115.607	70.20	− 51.13	1221.45	25.94
2,4-dimethyl-hexane	109.429	68.50	− 52.44	1220.15	25.23
2,5-dimethyl-hexane	109.103	68.60	− 53.21	1219.37	24.54
3,3-dimethyl-hexane	111.969	68.50	− 52.61	1219.97	26.19
3,4-dimethyl-hexane	117.725	70.20	− 50.91	1221.68	26.57
3-ethyl-2-methyl-pentane	115.450	69.70	− 50.48	1222.11	26.65
3-ethyl-3-methyl-pentane	118.259	69.30	− 51.38	1221.20	27.71
2,2,3-trimethyl-pentane	109.841	67.30	− 52.61	1219.98	26.94
2,2,4-trimethyl-pentane	99.238	64.87	− 53.57	1219.01	25.34
2,3,3-trimethyl-pentane	114.760	68.10	− 51.73	1220.86	27.83
2,3,4-trimethyl-pentane	113.467	68.37	− 51.97	1220.61	26.94
2,2,3,3-tetramethyl-butane	106.470	66.20	− 53.99	1218.51	28.30

**Fig. 1:**
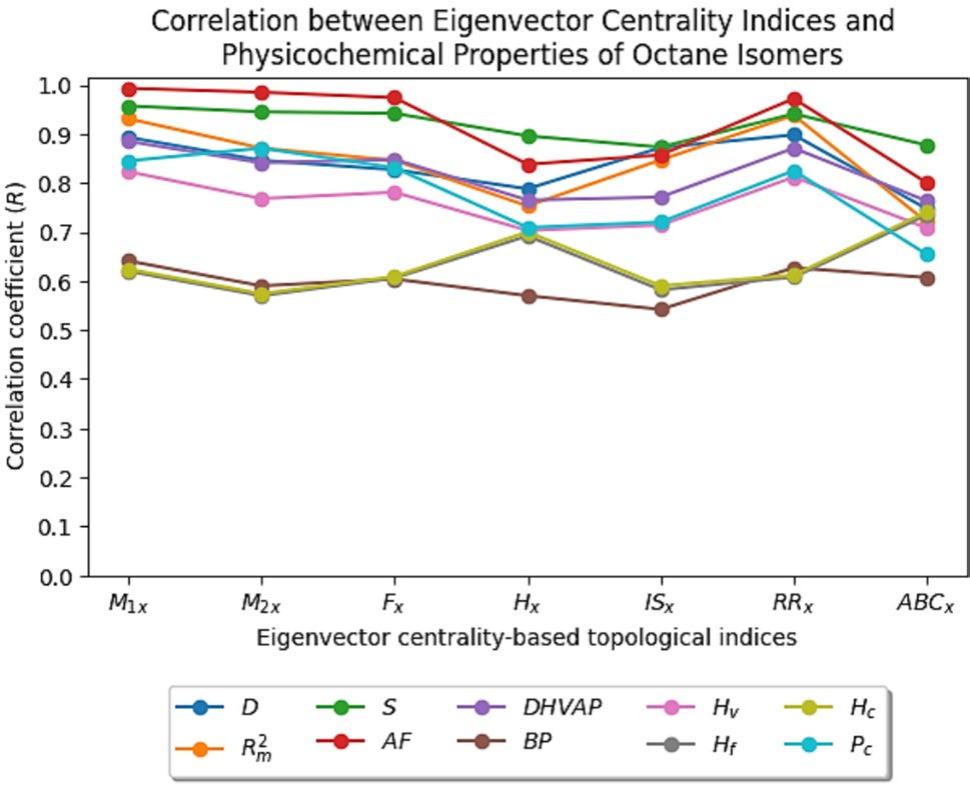
The correlation coefficient values of the eigenvector centrality-based topological indices with the physicochemical properties of octane isomers.

In this article, quadratic regression is employed for QSPR modeling due to its effectiveness in capturing non-linear relationships between the molecular descriptors and physicochemical properties. Preliminary analysis revealed that the relationship between the proposed eigenvector centrality-based indices and physicochemical properties is non-linear, following a parabolic trend rather than a more complex higher-order pattern. As a result, the quadratic regression yields higher correlation coefficients and lower standard errors compared to both linear and cubic regression models. The general quadratic regression equation for the eigenvector centrality-based indices is given by

$$P={c}_{1}{\left(TI\right)}^{2}+{c}_{2}\left(TI\right)+{c}_{3}$$

where $P$ is the physicochemical property, $TI$ is the eigenvector centrality-based topological index and ${c}_{1},{c}_{2}$ and ${c}_{3}$ are constants. The quadratic QSPR regression equations for all the desired properties are constructed using SPSS (Statistical Package for Social Sciences) software. These equations, along with their corresponding statistical parameters (correlation coefficient $(R)$, coefficient of determination $\left( {R}^{2}\right),$ standard error $\left( S.E\right)$, Fisher statistic $(F)$, significance value $(p)$) are tabulated in Tables 22, 23, 24, 25, 26, 27, 28, 29, 30 and 31. In each of these tables, the most significant quadratic regression equation, along with its corresponding statistics, is highlighted. The graphical representation of the correlation coefficient ($R$) values between the eigenvector centrality-based indices and the physicochemical properties is shown in Fig. 1.

**Table 22:** The quadratic QSPR regression equation with its corresponding values of statistical parameters (${R, R}^{2}, S.E, F, p$) for $D$.

TI	Quadratic regression equation	Statistics				
		$R$	${R}^{2}$	$S.E$	$F$	$p$
${{M}_{1}}_{x}$	$D=0.23{{M}_{1}^{2}}_{x}-2.41{{M}_{1}}_{x}+7.08$	0.893	0.798	0.014	29.624	0.000
${{M}_{2}}_{x}$	$D=4.72{{M}_{2}^{2}}_{x}-9.51{{M}_{2}}_{x}+5.49$	0.846	0.716	0.017	18.936	0.000
${F}_{x}$	$D=0.23{F}_{x}^{2}-1.02{F}_{x}+1.85$	0.827	0.684	0.018	16.215	0.000
${H}_{x}$	$D=(5E-3){H}_{x}^{2}-0.21{H}_{x}+2.88$	0.788	0.621	0.019	12.281	0.001
$I{S}_{x}$	$D=24.85I{S}_{x}^{2}-61.75I{S}_{x}+39.06$	0.872	0.760	0.015	23.760	0.000
${\varvec{R}}{{\varvec{R}}}_{{\varvec{x}}}$	${\varvec{D}}=2.05{\varvec{R}}{{\varvec{R}}}_{{\varvec{x}}}^{2}-10.54{\varvec{R}}{{\varvec{R}}}_{{\varvec{x}}}+14.23$	**0.898**	**0.807**	**0.014**	**31.320**	**0.000**
$AB{C}_{x}$	$D=\left(2.32E-3\right)AB{C}_{x}^{2}-0.12AB{C}_{x}+2.19$	0.747	0.559	0.021	9.493	0.002

**Table 23:** The quadratic QSPR regression equation with its corresponding values of statistical parameters (${R, R}^{2}, S.E, F, p$) for ${R}_{m}^{2}$.

TI	Quadratic regression equation	Statistics				
		$R$	${R}^{2}$	$S.E$	$F$	$p$
${{M}_{1}}_{x}$	${R}_{m}^{2}=0.9{{M}_{1}^{2}}_{x}-10.52{{M}_{1}}_{x}+32.17$	0.931	0.866	0.071	48.533	0.000
${{M}_{2}}_{x}$	${R}_{m}^{2}=15.44{{M}_{2}^{2}}_{x}-35.06{{M}_{2}}_{x}+21.37$	0.871	0.759	0.095	23.633	0.000
${F}_{x}$	${R}_{m}^{2}=0.84{F}_{x}^{2}-4.67{F}_{x}+7.93$	0.846	0.716	0.103	18.886	0.000
${H}_{x}$	${R}_{m}^{2}=\left(-7.4E-3\right){H}_{x}^{2}+0.38{H}_{x}-2.96$	0.753	0.567	0.127	9.817	0.002
$I{S}_{x}$	${R}_{m}^{2}=66.89I{S}_{x}^{2}-1.74E2I{S}_{x}+1.15E2$	0.847	0.717	0.103	18.990	0.000
${\varvec{R}}{{\varvec{R}}}_{{\varvec{x}}}$	${{\varvec{R}}}_{{\varvec{m}}}^{2}=8.2{\varvec{R}}{{\varvec{R}}}_{{\varvec{x}}}^{2}-45{\varvec{R}}{{\varvec{R}}}_{{\varvec{x}}}+63.19$	**0.938**	**0.880**	**0.067**	**54.908**	**0.000**
$AB{C}_{x}$	${R}_{m}^{2}=\left(-5.69E-3\right)AB{C}_{x}^{2}+0.33AB{C}_{x}-2.92$	0.720	0.518	0.134	8.065	0.004

**Table 24:** The quadratic QSPR regression equation with its corresponding values of statistical parameters (${R, R}^{2}, S.E, F, p$) for $S$.

TI	Quadratic regression equation	Statistics				
		$R$	${R}^{2}$	$S.E$	$F$	$p$
${{{\varvec{M}}}_{1}}_{{\varvec{x}}}$	${\varvec{S}}=-8.11{{{\varvec{M}}}_{1}^{2}}_{{\varvec{x}}}+71.14{{{\varvec{M}}}_{1}}_{{\varvec{x}}}-41.22$	**0.957**	**0.915**	**1.401**	**80.855**	**0.000**
${{M}_{2}}_{x}$	$S=-3.08E2{{M}_{2}^{2}}_{x}+5.67E2{{M}_{2}}_{x}-1.49E2$	0.945	0.894	1.568	63.071	0.000
${F}_{x}$	$S=-13.8{F}_{x}^{2}+50.37{F}_{x}+65.14$	0.942	0.887	1.617	58.829	0.000
${H}_{x}$	$S=-0.84{H}_{x}^{2}+35.3{H}_{x}-2.62E2$	0.896	0.803	2.137	30.479	0.000
$I{S}_{x}$	$S=-1.77E3I{S}_{x}^{2}+4.31E3I{S}_{x}-2.51E3$	0.873	0.762	2.347	23.982	0.000
$R{R}_{x}$	$S=-72.38R{R}_{x}^{2}+3.28E2R{R}_{x}-2.58E2$	0.941	0.886	1.626	58.112	0.000
$AB{C}_{x}$	$S=-0.44AB{C}_{x}^{2}+22.2AB{C}_{x}-1.71E2$	0.877	0.769	2.310	25.011	0.000

**Table 25:** The quadratic QSPR regression equation with its corresponding values of statistical parameters (${R, R}^{2}, S.E, F, p$) for $AF$.

TI	Quadratic regression equation	Statistics				
		$R$	${R}^{2}$	$S.E$	$F$	$p$
${{{\varvec{M}}}_{1}}_{{\varvec{x}}}$	${\varvec{A}}{\varvec{F}}=\left(1.44{\varvec{E}}-3\right){{{\varvec{M}}}_{1}^{2}}_{{\varvec{x}}}-0.16{{{\varvec{M}}}_{1}}_{{\varvec{x}}}+1.17$	**0.993**	**0.986**	**0.004**	**533.129**	**0.000**
${{M}_{2}}_{x}$	$AF=-{{M}_{2}^{2}}_{x}+1.44{{M}_{2}}_{x}-0.08$	0.985	0.971	0.006	252.463	0.000
${F}_{x}$	$AF=-0.04{F}_{x}^{2}+0.03{F}_{x}+0.47$	0.974	0.949	0.009	139.074	0.000
${H}_{x}$	$AF=\left(-4.47E-3\right){H}_{x}^{2}+0.19{H}_{x}-1.74$	0.838	0.703	0.021	17.711	0.000
$I{S}_{x}$	$AF=-4.99I{S}_{x}^{2}+11.33I{S}_{x}-6.02$	0.857	0.735	0.019	20.794	0.000
$R{R}_{x}$	$AF=0.04R{R}_{x}^{2}-0.65R{R}_{x}+1.74$	0.972	0.944	0.009	127.144	0.000
$AB{C}_{x}$	$AF=\left(-2.4E-3\right)AB{C}_{x}^{2}+0.13AB{C}_{x}-1.27$	0.800	0.640	0.023	13.348	0.000

**Table 26:** The quadratic QSPR regression equation with its corresponding values of statistical parameters (${R, R}^{2}, S.E, F, p$) for $DHVAP$.

TI	Quadratic regression equation	Statistics				
		$R$	${R}^{2}$	$S.E$	$F$	$p$
${{{\varvec{M}}}_{1}}_{{\varvec{x}}}$	${\varvec{D}}{\varvec{H}}{\varvec{V}}{\varvec{A}}{\varvec{P}}=0.69{{{\varvec{M}}}_{1}^{2}}_{{\varvec{x}}}-8.95{{{\varvec{M}}}_{1}}_{{\varvec{x}}}+37.38$	**0.885**	**0.784**	**0.190**	**27.170**	**0.000**
${{M}_{2}}_{x}$	$DHVAP=6.37{{M}_{2}^{2}}_{x}-19.35{{M}_{2}}_{x}+22.38$	0.841	0.708	0.220	18.195	0.000
${F}_{x}$	$DHVAP=0.37{F}_{x}^{2}-3.11{F}_{x}+14.46$	0.848	0.720	0.216	19.281	0.000
${H}_{x}$	$DHVAP=-0.05{H}_{x}^{2}+2.33{H}_{x}-15.33$	0.765	0.586	0.263	10.615	0.001
$I{S}_{x}$	$DHVAP=-34.73I{S}_{x}^{2}+75.32I{S}_{x}-30.7$	0.771	0.595	0.260	11.023	0.001
$R{R}_{x}$	$DHVAP=5.73R{R}_{x}^{2}-34.18R{R}_{x}+59.28$	0.871	0.758	0.201	23.525	0.000
$AB{C}_{x}$	$DHVAP=-0.03AB{C}_{x}^{2}+1.58AB{C}_{x}-10.59$	0.763	0.582	0.264	10.444	0.001

**Table 27:** The quadratic QSPR regression equation with its corresponding values of statistical parameters (${R, R}^{2}, S.E, F, p$) for $BP$.

TI	Quadratic regression equation	Statistics				
		$R$	${R}^{2}$	$S.E$	$F$	$p$
${{{\varvec{M}}}_{1}}_{{\varvec{x}}}$	${\varvec{B}}{\varvec{P}}=18.79{{{\varvec{M}}}_{1}^{2}}_{{\varvec{x}}}-2.21{\varvec{E}}2{{{\varvec{M}}}_{1}}_{{\varvec{x}}}+7.61{\varvec{E}}2$	**0.641**	**0.411**	**4.997**	**5.238**	**0.019**
${{M}_{2}}_{x}$	$BP=3.6E2{{M}_{2}^{2}}_{x}-8.16E2{{M}_{2}}_{x}+5.72E2$	0.590	0.349	5.256	4.014	0.004
${F}_{x}$	$BP=17.03{F}_{x}^{2}-96.83{F}_{x}+2.47E2$	0.604	0.365	5.191	4.304	0.033
${H}_{x}$	$BP=-0.83{H}_{x}^{2}+34.48{H}_{x}-2.41E2$	0.570	0.325	5.351	3.608	0.053
$I{S}_{x}$	$BP=-2.93E2I{S}_{x}^{2}+6.02E2I{S}_{x}-1.8E2$	0.542	0.294	5.473	3.117	0.074
$R{R}_{x}$	$BP=1.48E2R{R}_{x}^{2}-8.21E2R{R}_{x}+1.25E3$	0.627	0.393	5.072	4.865	0.024
$AB{C}_{x}$	$BP=-0.49AB{C}_{x}^{2}+24.15AB{C}_{x}-1.82E2$	0.607	0.369	5.173	4.384	0.032

**Table 28:** The quadratic QSPR regression equation with its corresponding values of statistical parameters (${R, R}^{2}, S.E, F, p$) for ${H}_{v}$.

TI	Quadratic regression equation	Statistics				
		$R$	${R}^{2}$	$S.E$	$F$	$p$
${{{\varvec{M}}}_{1}}_{{\varvec{x}}}$	${{\varvec{H}}}_{{\varvec{v}}}=4.87{{{\varvec{M}}}_{1}^{2}}_{{\varvec{x}}}-60.15{{{\varvec{M}}}_{1}}_{{\varvec{x}}}+2.52{\varvec{E}}2$	**0.823**	**0.678**	**1.224**	**15.788**	**0.000**
${{M}_{2}}_{x}$	${H}_{v}=55.68{{M}_{2}^{2}}_{x}-1.45E2{{M}_{2}}_{x}+1.6E2$	0.768	0.589	1.382	10.761	0.001
${F}_{x}$	${H}_{v}=3.11{F}_{x}^{2}-21.41{F}_{x}+1.03E2$	0.781	0.609	1.348	11.705	0.001
${H}_{x}$	${H}_{v}=-0.28{H}_{x}^{2}+11.67{H}_{x}-52.91$	0.703	0.494	1.534	7.334	0.006
$I{S}_{x}$	${H}_{v}=-39.39I{S}_{x}^{2}+39.88I{S}_{x}+81.03$	0.714	0.509	1.511	7.782	0.005
$R{R}_{x}$	${H}_{v}=41.6R{R}_{x}^{2}-2.38E2R{R}_{x}+4.08E2$	0.812	0.659	1.260	14.497	0.000
$AB{C}_{x}$	${H}_{v}=-0.16AB{C}_{x}^{2}+7.96AB{C}_{x}-30.1$	0.708	0.501	1.523	7.544	0.005

**Table 29:** The quadratic QSPR regression equation with its corresponding values of statistical parameters (${R, R}^{2}, S.E, F, p$) for ${H}_{f}$.

TI	Quadratic regression equation	Statistics				
		$R$	${R}^{2}$	$S.E$	$F$	$p$
${{M}_{1}}_{x}$	${H}_{f}=0.87{{M}_{1}^{2}}_{x}-12.72{{M}_{1}}_{x}-8.32$	0.620	0.385	1.045	4.696	0.026
${{M}_{2}}_{x}$	${H}_{f}=-4.81{{M}_{2}^{2}}_{x}-3.32{{M}_{2}}_{x}-43.09$	0.570	0.325	1.094	3.616	0.052
${F}_{x}$	${H}_{f}=-0.4{F}_{x}^{2}-1.11{F}_{x}-46.78$	0.606	0.367	1.060	4.345	0.032
${H}_{x}$	${H}_{f}=-0.25{H}_{x}^{2}+10.01{H}_{x}-1.53E2$	0.692	0.479	0.962	6.900	0.008
$I{S}_{x}$	${H}_{f}=-4E2I{S}_{x}^{2}+9.78E2I{S}_{x}-6.49E2$	0.582	0.339	1.084	3.840	0.045
$R{R}_{x}$	${H}_{f}=3.89R{R}_{x}^{2}-29.84R{R}_{x}-0.5$	0.608	0.369	1.058	4.389	0.032
${\varvec{A}}{\varvec{B}}{{\varvec{C}}}_{{\varvec{x}}}$	${{\varvec{H}}}_{{\varvec{f}}}=-0.14{\varvec{A}}{\varvec{B}}{{\varvec{C}}}_{{\varvec{x}}}^{2}+6.59{\varvec{A}}{\varvec{B}}{{\varvec{C}}}_{{\varvec{x}}}-1.31{\varvec{E}}2$	**0.735**	**0.540**	**0.904**	**8.790**	**0.003**

**Table 30:** The quadratic QSPR regression equation with its corresponding values of statistical parameters (${R, R}^{2}, S.E, F, p$) for ${H}_{c}$.

TI	Quadratic regression equation	Statistics				
		$R$	${R}^{2}$	$S.E$	$F$	$p$
${{M}_{1}}_{x}$	${H}_{c}=0.69{{M}_{1}^{2}}_{x}-10.8{{M}_{1}}_{x}+1.26E3$	0.624	0.389	1.049	4.782	0.025
${{M}_{2}}_{x}$	${H}_{c}=-8.3{{M}_{2}^{2}}_{x}+3.79{{M}_{2}}_{x}+1.23E3$	0.574	0.329	1.099	3.685	0.050
${F}_{x}$	${H}_{c}=-0.57{F}_{x}^{2}-0.32{F}_{x}+1.23E3$	0.609	0.371	1.064	4.427	0.031
${H}_{x}$	${H}_{c}=-0.25{H}_{x}^{2}+10.19{H}_{x}+1.12E3$	0.700	0.490	0.959	7.194	0.006
$I{S}_{x}$	${H}_{c}=-4.21E2I{S}_{x}^{2}+1.03E3I{S}_{x}+5.9E2$	0.590	0.349	1.083	4.013	0.040
$R{R}_{x}$	${H}_{c}=2.24R{R}_{x}^{2}-21.27R{R}_{x}+1.26E3$	0.612	0.374	1.062	4.487	0.030
${\varvec{A}}{\varvec{B}}{{\varvec{C}}}_{{\varvec{x}}}$	${{\varvec{H}}}_{{\varvec{c}}}=-0.14{\varvec{A}}{\varvec{B}}{{\varvec{C}}}_{{\varvec{x}}}^{2}+6.68{\varvec{A}}{\varvec{B}}{{\varvec{C}}}_{{\varvec{x}}}+1.14{\varvec{E}}3$	**0.741**	**0.549**	**0.901**	**9.139**	**0.003**

**Table 31:** The quadratic QSPR regression equation with its corresponding values of statistical parameters (${R, R}^{2}, S.E, F, p$) for ${P}_{c}$.

TI	Quadratic regression equation	Statistics				
		$R$	${R}^{2}$	$S.E$	$F$	$p$
${{M}_{1}}_{x}$	${P}_{c}=0.89{{M}_{1}^{2}}_{x}-5.57{{M}_{1}}_{x}+29.79$	0.845	0.715	0.687	18.779	0.000
${{{\varvec{M}}}_{2}}_{{\varvec{x}}}$	${{\varvec{P}}}_{{\varvec{c}}}=51.04{{{\varvec{M}}}_{2}^{2}}_{{\varvec{x}}}-87.23{{{\varvec{M}}}_{2}}_{{\varvec{x}}}+61.27$	**0.871**	**0.759**	**0.631**	**23.584**	**0.000**
${F}_{x}$	${P}_{c}=1.83{F}_{x}^{2}-4.89{F}_{x}+26.99$	0.831	0.691	0.715	16.738	0.000
${H}_{x}$	${P}_{c}=0.05{H}_{x}^{2}-2.68{H}_{x}+57.75$	0.709	0.503	0.906	7.593	0.005
$I{S}_{x}$	${P}_{c}=36.94I{S}_{x}^{2}-57.35I{S}_{x}+39.86$	0.720	0.518	0.893	8.056	0.004
$R{R}_{x}$	${P}_{c}=4.52R{R}_{x}^{2}-11.44R{R}_{x}+25.08$	0.825	0.681	0.726	15.995	0.000
$AB{C}_{x}$	${P}_{c}=0.03AB{C}_{x}^{2}-1.63AB{C}_{x}+49.39$	0.654	0.427	0.973	5.594	0.015

In QSPR analysis, the statistical criteria considered for assessing goodness of fit between the properties of octane isomers and the computed eigenvector centrality indices are $max(R), max({R}^{2}), max(F), minimal(S. E)$ and $p<0.05$*.* From Tables 22, 23, 24, 25, 26, 27, 28, 29, 30 and 31, it can be observed that all the constructed quadratic QSPR regression equations yield good correlation with $R>0.5$ and $minimal (S.E)$.

The most prominent indices for exploring the physicochemical properties of octane isomers using quadratic QSPR regression equations are given below.

- ${{M}_{1}}_{x}$ exhibits excellent correlation coefficients of 0.957, 0.993, 0.885, 0.641 and 0.823 with entropy $\left(S\right),$ acentric factor $(AF),$ standard enthalpy of vaporization $\left(DHVAP\right)$ and enthalpy of vaporization ${(H}_{v})$, respectively.
- ${{M}_{1}}_{x}$ has a better correlation with boiling point $(BP)$ than the other six indices, with a correlation coefficient of 0.641.
- ${{M}_{2}}_{x}$ has a high correlation coefficient of 0.871 with critical point ${(P}_{c})$.
- $R{R}_{x}$ yields strong correlation coefficient of 0.898 and 0.938 with density $(D)$ and mean radius ${(R}_{m}^{2})$ respectively.
- $AB{C}_{x}$ shows good correlations of 0.735 and 0.741 with enthalpy of formation ${(H}_{f})$ and enthalpy of combustion ${(H}_{c})$, respectively.

**Fig. 2:**
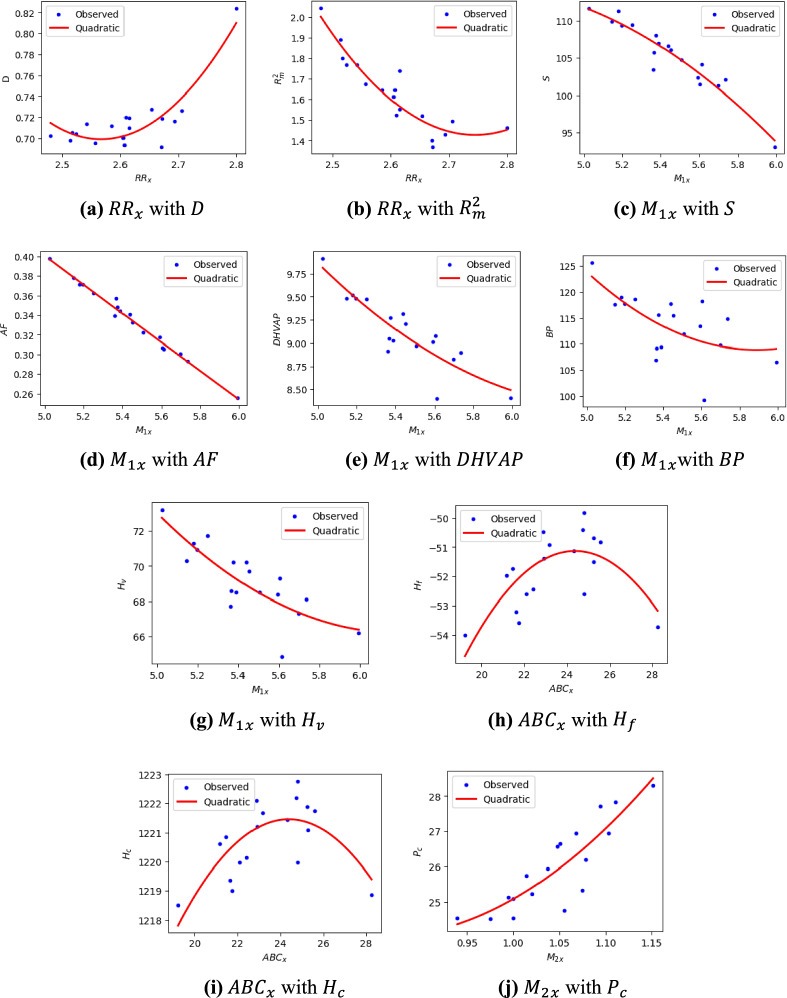
Scattering plots of the most significant quadratic QSPR regression equations (**a**) $R{R}_{x}$ with $D$, (**b**) $R{R}_{x}$ with ${R}_{m}^{2}$, (**c**)${{M}_{1}}_{x}$ with $S$, (**d**) ${{M}_{1}}_{x}$ with $AF$, (**e**) ${{M}_{1}}_{x}$ with $DHVAP$, (**f**) ${{M}_{1}}_{x}$ with $BP$ (**g**) ${{M}_{1}}_{x}$ with ${H}_{v}$, (**h**) $AB{C}_{x}$ with ${H}_{f}$, (**i**) ${ABC}_{x}$ with ${H}_{c}$ and (**j**) ${{M}_{2}}_{x}$ with ${P}_{c}$.

The scatter plots related to the most significant quadratic QSPR regression equations highlighted in Tables 22, 23, 24, 25, 26, 27, 28, 29, 30 and 31 are displayed in Fig. 2 to more visibly demonstrate the statistical significance of the introduced indices. It is vital to recognize the effectiveness of these novel indices. Like any other indices, their performance may depend on various factors, including the size and diversity of the dataset, the modeling techniques employed, and the specific properties being explored. Consequently, further in-depth research is required to fully understand the capabilities and limitations of these indices when exploring the physicochemical properties of various chemical compounds.

### Y-randomization test

**Fig. 3:**
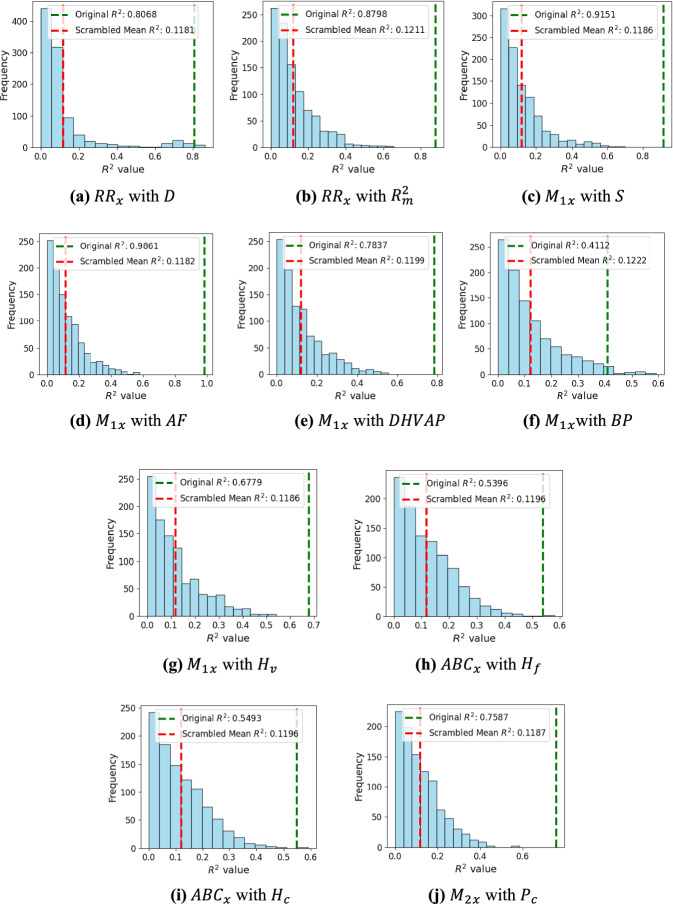
Y-randomization ${R}^{2}$ distribution plot showing the difference between the original ${R}^{2}$ and scrambled mean ${R}^{2}$ for (**a**) $R{R}_{x}$ with $D$, (**b**) $R{R}_{x}$ with ${R}_{m}^{2}$, (**c**)${{M}_{1}}_{x}$ with $S$, (**d**) ${{M}_{1}}_{x}$ with $AF$, (**e**) ${{M}_{1}}_{x}$ with $DHVAP$, (**f**) ${{M}_{1}}_{x}$ with $BP$ (**g**) ${{M}_{1}}_{x}$ with ${H}_{v}$, (**h**) $AB{C}_{x}$ with ${H}_{f}$, (**i**) ${ABC}_{x}$ with ${H}_{c}$ and (**j**) ${{M}_{2}}_{x}$ with ${P}_{c}$.

In this research, the Y-randomization test is performed between the calculated eigenvector centrality-based indices (independent variables, X) and the experimental properties (dependent variable, Y). The test is implemented using Python programming, and the results are shown in Fig. 3, which illustrates the difference between the original *R*^2^ and scrambled *R*^2^ scores over 1000 iterations.

The results of the Y-randomization test strongly support the validity of the quadratic regression models for predicting the physicochemical properties of octane isomers. For the majority of properties, the original *R*^2^ values are significantly higher than the scrambled mean *R*^2^, with differences ranging from 0.2890 to 0.8679. This suggests that the models are not overfitted and that the relationship between the indices and the properties are statistically significant and robust. Overall, the test provides strong evidence that the regression models developed in this research effectively capture meaningful relationships and are not merely the result of random chance.

### Chi-square test for goodness of fit

The chi-square test for goodness of fit is conducted at the 5% and 1% significance levels to evaluate whether the proposed regression equations accurately fit the physicochemical properties of octane isomers. Below is a concise explanation and inference based on the test results:

- ${\varvec{H}}$_**0**_: The regression equation accurately predicts the physicochemical property.
- ${\varvec{H}}$_**1**_: The regression equation does not accurately predict the physicochemical property.

**Fig. 4:**
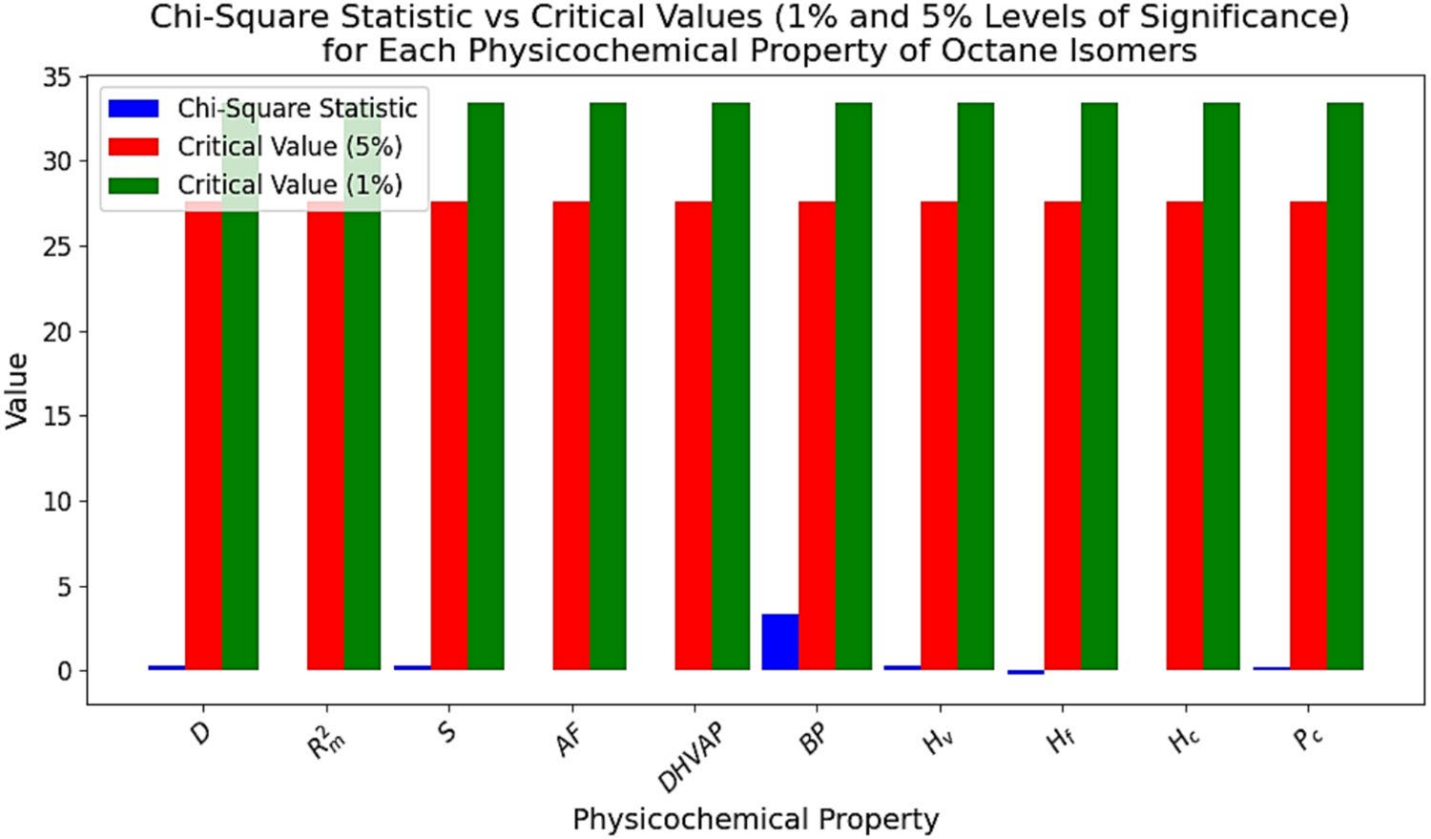
The bar plot of the chi-square statistic vs critical values at 1% and 5% levels of significance for the physicochemical properties of octane isomers.

The chi-square statistic for each physicochemical property is relatively small (ranging from 0.0009 to 3.3523), indicating minimal deviation between observed and expected values. Since the chi-square statistic consistently falls below the critical values at both the 1% and 5% significance levels for all properties, the differences between observed and expected values are negligible. The bar plot of the chi-square statistic vs critical values at 1% and 5% levels of significance for the physicochemical properties of octane isomers is presented in Fig. 4. With $p$-values close to 1.0, there is no evidence to reject the null hypothesis, suggesting that the equation fits the physicochemical properties of octane isomers effectively across all tested properties.

Testing the octane isomer properties and the proposed indices demonstrates that the introduced indices are effective in developing predictive models. By confirming the statistical significance of the observed relationships, it is ensured that the conclusions drawn from the model are robust and reliable, paving the way for their application in real-world chemical property predictions.

## Conclusion

In this article, seven new topological indices based on the eigenvector centrality of molecular graphs were introduced, namely First eigenvector centrality index $({M}_{{1}_{x}})$, Second eigenvector centrality index $({M}_{{2}_{x}})$, Forgotten eigenvector centrality index ${(F}_{x})$, Harmonic eigenvector centrality index ${(H}_{x})$, Inverse sum eigenvector centrality index $(I{S}_{x})$, Reciprocal Randić eigenvector centrality index $(R{R}_{x})$ and Atom-bond connectivity eigenvector centrality index $(AB{C}_{x})$. These indices were computed for eighteen octane isomers viz, $n$-octane, 2-methylheptane, 3-methylheptane, 4-methylheptane, 3-ethylhexane, 2,2-dimethylhexane, 2,3-dimethylhexane, 2,4-dimethylhexane, 2,5-dimethylhexane, 3,3-dimethylhexane, 3,4-dimethylhexane, 3-ethyl-2-methylpentane, 3-ethyl-3-methylpentane, 2,2,3-trimethylpentane, 2,2,4-trimethylpentane, 2,3,3-trimethylpentane, 2,3,4-trimethylpentane and 2,2,3,3-tetramethylbutane. Quadratic regression was employed in QSPR modeling to investigate the relationships between the proposed indices and various physicochemical properties, including density $(D)$, mean radius $({R}_{m}^{2})$, entropy $(S)$, acentric factor $(AF$), standard enthalpy of vaporization $(DHVAP)$, boiling point $(BP)$, enthalpy of vaporization $({H}_{v})$, enthalpy of formation $({H}_{f})$, enthalpy of combustion $({H}_{c})$ and critical pressure $({P}_{c})$.

The results indicate that the proposed indices exhibit a strong correlation with the target properties. The proposed eigenvector centrality-based indices demonstrate robust predictive performance, as validated by Y-randomization and chi-square tests, confirming the models’ statistical significance. This correlation underscores the applicability of eigenvector centrality-based indices beyond octane isomers, highlighting their reliability and effectiveness in capturing intricate molecular characteristics. Their strong predictive performance suggests promising QSPR and QSAR applications, including drug discovery targeting cancer and antiviral therapies. This work lays a solid foundation for future research, integrating eigenvector centrality into cheminformatics and pharmaceutical modeling.

## Data Availability

The molecular structures and datasets analyzed in this research are publicly available in the ChemSpider repository (https://www.chemspider.com/). Data analysis was conducted using the Statistical Package for the Social Sciences (SPSS) software (https://www.ibm.com/spss), while statistical tests were performed using Python programming.
